# Attitudes of Internal Medicine Nurses, Surgical Nurses and Midwives towards Reporting of Clinical Adverse Events

**DOI:** 10.3390/healthcare12010115

**Published:** 2024-01-03

**Authors:** Anna Majda, Michalina Majkut, Aldona Wróbel, Alicja Kamińska, Anna Kurowska, Agata Wojcieszek, Kinga Kołodziej, Krystian Barzykowski

**Affiliations:** 1 Laboratory of Theory and Fundamentals of Nursing, Institute of Nursing and Midwifery, Faculty of Health Sciences, Jagiellonian University Medical College, Michałowskiego 12 Street, 31-126 Krakow, Poland; anna.majda@uj.edu.pl (A.M.); aldona1.wrobel@uj.edu.pl (A.W.); alicja.kaminska@uj.edu.pl (A.K.); anna2.kurowska@uj.edu.pl (A.K.); agata.wojcieszek@uj.edu.pl (A.W.); kinga1.kolodziej@uj.edu.pl (K.K.); 2 Institute of Psychology, Jagiellonian University, Ingardena 6 Street, 30-060 Krakow, Poland

**Keywords:** adverse events, attitudes, nurses, midwives

## Abstract

Understanding the attitudes of medical staff contributes to shaping a culture of safety in health care. The aim of this study was the measurement of attitudes of nurses and midwives towards reporting clinical adverse events. Various research tools were used, including the Reporting of Clinical Adverse Events Scale (RoCAES; Polish: P-RoCAES), the Justice Sensitivity Inventory, the Feelings in Moral Situations Scale, the Perceived Stress at Work Scale and the Author’s Survey Questionnaire. The cross-sectional survey was conducted from October 2022 to April 2023. The study used assessment-based sampling. The study included 745 midwives and nurses working in internal medicine—surgical wards in nine hospitals in a large provincial city in Poland. One-way analysis of variance ANOVA, post hoc test (Fisher’s NIR), and r-Spearman correlation test were used. The level of significance (*p*) did not exceed 0.05. Respondents did not differ in terms of sensitivity to justice, moral feelings, and perceived stress at work, all of which variables were at moderate levels. Respondents’ attitudes towards reporting clinical adverse events in the P-RoCAES were positive (surgical nurses 71.10; internal medicine nurses 72.04; midwives 71.26; F(2.741) = 1.14, *p* = 0.319), especially those with a master’s degree, longer work experience and older age. Respondents with a master’s degree were most likely to perceive a benefit from reporting adverse events (P-RoCAES subscale) (F(2.737) = 8.45, *p* = 0.001). The longer employment tenure (F(3.716) = 4.63, *p* = 0.003) and having a master’s degree (F(2.737) = 3.10, *p* = 0.045) were associated with a higher feeling of guilt among the respondents (P-RoCAES subscale). The longer the participants worked, the more positive their attitude became towards the importance of transparency in procedures (F(2.741) = 3.56, *p* = 0.029), but the more negative their attitude was towards the benefits of reporting adverse events (P-RoCAES subscale) (r(686) = −0.08, *p* = 0.037). Individual attitudes of nurses and midwives as well as their age, length of service or education can influence the formation of a culture of safety in health care (including the reporting of clinical adverse events). Attitudes can motivate corrective action, can be reinforced and shaped by educational programs, good quality management and monitoring system solutions.

## 1. Introduction

### 1.1. Attitudes as an Object of Research Interest

Attitudes are a very important part of everyone’s life, reflecting their approach to the world and to particular areas of life. They are formed in the process of upbringing and are influenced by various factors. The formation of attitudes is a very complex and long-term process, which begins in early childhood. This process continues throughout life, including working life. It is easier to reinforce or form attitudes than to change them. When they need to be modified, it is easier to change weak views than strong, less enduring views than strongly internalized ones. A person’s attitude is generally a positive or negative attitude towards someone or something, reflected in beliefs, emotions, perception of surrounding reality and behavior [1]. Learning about attitudes concerns medical staff, including nurses and midwives, who need to form an attitude of professional response to clinical adverse events in the course of developing knowledge, skills and safe practices [2]. A wide variety of definitions of attitude can be found in the literature, and this is related to the fact that the concept has overtones in the terminology of many social sciences, such as psychology, pedagogy, sociology; the humanities, which includes philosophy [3]; or the health sciences, which includes nursing [4]. Attitudes are usually defined as the tendency to react positively or negatively to an object, person, or event. Attitudes are among the key concepts used to explain social behavior. According to the “Psychological Dictionary” [5], an attitude is a relatively permanent, dynamic organization of cognitive, emotional structures and behavioral patterns associated with a particular object, characterized by certain complexity, content, strength, durability, intensity, validity, and relevance. Attitudes themselves are a hypothetical construct, being unobservable directly, they can only be determined based on external indicators. Usually, these indicators refer to three types of reactions to the attitude object. Attitudes can be formulated first based on cognitive reactions (beliefs, messages), second, they can be determined based on affective reactions (feelings, evaluations attributed to objects), and third, based on behavioral reactions (intentions, tendencies and actions related to the object) [6,7]. This strand of thinking represents the so-called three-component approach to understanding attitudes [8,9]. It creates serious difficulties in the development of an attitude index, in the operationalization and construction of a measurement method. This is because three very different types of variables need to be measured. This issue is the subject of lively discussion in the field of attitude research. The main controversy is over the issue of the relationship between the three components and the strong tendency to point to one element as constitutive of attitudes. The emotional–motivational component is most often considered such an element. Newer accounts of attitudes allow us to expect great progress in understanding the relationship between attitudes and human behavior [1]. The subject of much work by social psychologists has been techniques for studying attitudes. They can be divided into techniques based on observation of behavior, attitude scales or less structured techniques (interview, questionnaires, projective methods). Among the most popular methods of constructing attitude scales are the Thurstone, Likert, Guttman techniques [10]. These scales are based on the evaluation of an object on a presented value scale. The attitude is then expressed by a certain number, the interpretation of which depends on the measurement technique adopted. The issue of attitude formation is very complex due to the fact that it involves different areas of the personality—intellect, emotions, behavior. The basic mechanism of attitude formation is direct or indirect contact with the attitude object. Different components of attitudes need to be influenced, e.g., intellectual—by providing knowledge about the attitude object; behavioral—by engaging in a particular activity; emotional—by arousing certain feelings, e.g., a sense of satisfaction. To shape attitudes, you need to be consistent in your message and credible in your choice of arguments [1].

### 1.2. Adverse Events as an Object of Research Interest

Adverse events, in the context of health care, are harm caused during or as a result of treatment, that is unrelated to the natural course of the disease, the patient’s condition or risk of occurrence [11]. Medical professionals’ knowledge of adverse events in the healthcare system is practically limited to medical causes, such as side effects of the drugs and procedures. Patient falls, bedsores, complications accompanying parenteral treatment and hospital-acquired infections are usually considered within this [12,13,14]. Causes outside the healthcare system, the ergonomic inadequacies of the system or the attitudes of medical staff towards reporting clinical adverse events are almost completely ignored. Adverse events have different effects on patients and organizations. They lead to temporary or permanent disability or even death. They are associated with high medical costs due to prolonged hospital stay, readmissions to hospital, unfavorable opinions in the media or legal action [15]. Adverse events are a key issue affecting patient safety in healthcare services. In order to create a safe environment for patients, it is important to assess the attitudes of nurses and midwives towards adverse event reporting. However, to the best of the authors’ knowledge, there are few reports in the Polish literature on the subject directly relating to the attitudes of nurses and midwives towards adverse events using standardized research tools [16]. Few studies on attitudes of medical professionals have appeared in the foreign literature, based on a scant number of standardized research tools. From the literature, we found several different version instruments (Medication Administration Error Reporting scale; Nursing Staff/Pharmacist/Medical Staff Questionnaire Regarding Error reporting; Hospital Survey on Patient Safety Culture; Incident Reporting Culture Questionnaires) for measuring nurses’ reporting status. One of the questionnaires used worldwide to measure attitudes of health care professionals towards adverse events is the Reporting of Clinical Adverse Events Scale (RoCAES)—constructed in 2008 in the United Kingdom, by Wilson B., Bekker H.L., Fylan F. It is characterized by a high reliability index (0.83) and relevance, confirmed by German (RoCAES-D, 0.79), Chinese (C-RoCAES, 0.85) and Polish (P-RoCAES, 0.82) studies [17,18,19,20]. The issues raised in the article are important because there is a need to take appropriate preventive measures to minimize the causes and occurrence of adverse events, thereby increasing patient safety in health care. According to Nowak–Musiej, adverse events affect 4 in 10 patients in outpatient care (80% of which could be prevented) and 6% of hospitalized patients (in OECD countries). The costs incurred to remedy the effects of adverse events are 0.2%–16.5% of public hospital expenditures [21]. Description of the attitudes of nurses and midwives towards reporting adverse events can fill the gaps in the Polish publishing market, contribute to building a safe work environment in medical entities, and develop preventive measures—educational by promoting a culture of reporting adverse events without mutual accusations and looking for someone to blame (no blame/fair blame).

The purpose of this study was to measure the attitudes of nurses working in internal medicine departments and surgical departments, as well as midwives, working in obstetrics departments, towards adverse events. These groups were chosen because adverse events occur in these wards during hospitalization due to their characteristics. In addition, the relationship of attitudes towards clinical adverse events with selected sociodemographic variables, such as seniority, age, education, are analyzed.

## 2. Materials and Methods

### 2.1. Design and Data Collection

The cross-sectional survey was conducted from October 2022 to April 2023. The inclusion criterion was nurses working in the hospital in internal medicine departments and surgical departments, as well as midwives, working in obstetrics departments, while the exclusion criterion was midwives and nurses working in primary care, hospital administration.

The survey covered nurses working in internal medicine and surgical departments, as well as midwives in 9 hospitals (out of 12 existing), in a major provincial city in Poland, which employed about 3100 nurses and midwives. Approvals were obtained from the directors to conduct the study. The directors of 3 hospitals did not give their consent—there were about 580 nurses and midwives employed there. A total of 3100 survey sheets were distributed. Of the 1100 sheets returned, 745 were correct completions (i.e., without irregularities or missing data), which were included in further analyses. The return ratio was 35.48%. The sample was representative of a broader spectrum of nurses and midwives. With this sample size and number of nurses and midwives, the margin of error was 3.59% (confidence level 0.95% and proportion 0.50). The study used judgmental sampling. This type of purposive sampling includes individuals selected for inclusion in the study based on the researcher’s professional judgment. It is the opposite of probability sampling, in which individuals are drawn with some probability (e.g., randomly) from the population of interest. 

Questionnaires were available on paper. They were distributed to the individual hospitals and wards by the project manager and then collected after one week from the nurses and midwives who completed them and left them with the ward nurses/midwives in locked envelopes. The data collected was entered by the research assistants in an Excel file/form. Permission was obtained from the authors to use all questionnaires of the research tools used in the study.

### 2.2. Measurement Tools

The study used a diagnostic survey and estimation method, as well as a survey and scaling technique. The study used 5 research tools.

Reporting of Clinical Adverse Events Scale (RoCAES; Polish: P-RoCAES) by Wilson B., Bekker H.L., Fylan F. It consisted of 25 statements assessing attitudes towards reporting clinical adverse events. It included five dimensions/subscales: feelings of guilt (6 items), perceived criteria for identifying events to report (6 items), perceived expectations of colleagues (6 items), perceived benefits of reporting adverse events (5 items), and perceived transparency of reporting procedures (2 items). Respondents provided answers according to the Likert scale (1–4), where 1 meant strongly agree and 4 meant strongly disagree. The values of all responses were then summed, with the scores for 16 statements reversed (see below). The overall score could range from 25 (very positive attitude towards reporting) to 100 (very negative attitude) points [17]. The Polish study, authored by Majkut, Majda and Barzykowski, the RoCAES was given the acronym P-RoCAES, found reliability (internal consistency: Cronbach’s alpha 0.82; McDonald’s omega 0.80), and theoretical relevance. The tool was found to be valid and reliable for assessing the attitudes of medical professionals (nurses) and medical students (nursing). As in the original 16 statements, the scoring was reversed. Because the original version used a non-intuitive scale where 1 meant strongly agree and 4 meant strongly disagree, in this study it was decided to modify the Likert scale (1–4) so that 1 meant strongly disagree and 4 meant strongly agree. Consequently, the overall scores ranged from 25 (very negative attitudes towards adverse events) to 100 points (very positive attitudes towards adverse events) [20].

The Author’s Survey Questionnaire includes 15 questions that relate to the occurrence and reporting of adverse events, reasons for not reporting, experiences with reporting, the body for monitoring adverse events (this information will be used as part of a separate research subject and therefore the results are not presented in this article), and metrics.

In addition, the study chose to use three tools: (1) the Justice Sensitivity Inventory, (2) the Feelings in Moral Situations Scale, and (3) the Perceived Stress at Work Scale. These tools were used to verify the assumption of comparability between groups of respondents working in the three types of departments (internal medicine, surgical, obstetric) in terms of variables that could be relevant to the formation of attitudes towards adverse events. For example, guilt and pride have constructive motivational potential for attitude change [22]. On the other hand, sensitivity to justice is a factor that may influence the respondents’ attitudes towards situations involving unfair treatment of others and non-compliance with ethical norms, especially in interpersonal relations [23]. Finally, stress at work carries the risk of mistakes, adverse events, and reduces efficiency and conscientiousness in performing professional duties [24]. These tools are discussed in detail below, although the results obtained through them will not be presented in detail or discussed, as they will be used as part of a separate research subject.

The Justice Sensitivity Inventory by Schmitt M., Gollwitzer M., Maes J., Arbach D., Polish adaptation: Chudzicka-Czupała A. (Cronbach’s alpha: subscale perspective of victim 0.87; subscale perspective of witness 0.82; subscale perspective of beneficiary 0.92); allows the assessment of reactions to one’s own and others’ acts in the context of unfair treatment. Each part/subscale contained 10 statements relating to reactions to injustice from the perspective of the victim, witness, unauthorized beneficiary, perpetrator, and one question about the frequency of experiencing the listed situations. The digits selected by the survey participant for each part were summed, and then the average value was calculated. A higher number of points scored in each part meant a proportionally higher sensitivity to justice [23].

The Feelings in Moral Situations Scale by Strus W. (Cronbach’s alpha for each subscale ranged from 0.53 to 0.89) makes it possible to examine the predisposition to experiencing various emotions. It compiles feelings accompanying non-compliance with important moral principles (part A—26 terms) and following these principles (part B—25 terms). Scale A deals with transgressing norms, while scale B deals with realizing them [22].

The Perceived Stress at Work Scale by Chirkowska-Smolak T., Grobelny J. (Cronbach’s alpha 0.85; Sperman’s correlation 0.17; 0.39) allows assessment of an employee’s interaction with the work environment. It consists of 10 questions that addressed feelings and thoughts of the past month about work life and its accompanying difficulties. The scores for the four questions were reversed, and then all the point values (0–4) were summed [24].

### 2.3. Ethical Considerations

The research tools were selected in such a way that their content did not violate the welfare of the survey participants. Respondents were presented with the purpose of the study. They were informed that the survey was voluntary and anonymous, preceded by consent. The survey participants were aware that they would be able to withdraw at any stage of the survey and refuse to complete the prepared tools without suffering any consequences for doing so. The respondents were aware that the results would be used only for scientific purposes and would be subject to anonymization—they would be made public in a form that excluded the identification of the respondents. In conducting the study, the information was contained in the following: (1) the World Medical Association’s Declaration of Helsinki; (2) the Data Protection Act of May 10, 2018; (3) the Code of Ethics for Research Workers. The study was conducted within the framework of statutory project NR43/DBS/000233. The study received approval from the Bioethics Committee of Jagiellonian University Medical College in Kraków No. 1072.6120.214.2022.

### 2.4. Data Analysis

The collected data were subjected to statistical analysis. For this purpose, the program Statistica (version 13), one-way ANOVA analysis of variance with a grouping variable of ward type (internal medicine wards, surgical wards, maternity wards), and age category or education, were used. The level of significance (p) did not exceed 0.05. Differences between groups were counted using a post hoc test (Fisher’s NIR). Correlation was analyzed using r-Spearman.

## 3. Results

First, in comparative analyses of three groups—internal medicine nurses, surgical nurses, midwives—in terms of justice sensitivity, moral feelings and their perceived stress at work were presented. This made it possible to verify the extent to which the groups were comparable in this regard. Second, we presented an analysis of the differences in the P-RoCAES questionnaire scores between the three groups of participants: internal medicine nurses, surgical nurses, and midwives. Finally, the relationship of the results in the P-RoCAES questionnaire with selected sociodemographic variables, such as, age, length of service, and education, were presented.

### 3.1. Individuals Surveyed

The analysis finally included 745 participants, of whom 95.84% (714 participants) were women, 4.16% (31 participants) were men. The age of the participants ranged between 21 and 67, with the percentage of participants (and numbers) in each age range as follows: (a) 21–30 years—26.44% (197 participants); (b) 31–40 years—18.26% (136 participants); (c) 41–50 years—25.37% (189 participants); (d) 51–60 years—26.71% (199 participants); (e) 61–67 years—3.22% (24 participants). Nurses of internal medicine wards accounted for 61.34% (457 participants), of surgical wards 22.55% (168 participants), while midwives accounted for 16.11% (120 participants). A number of 33.96% (253 participants) had a bachelor’s degree, 49.53% (369 participants) had a master’s degree, 15.97% (119 participants) declared they had a diploma (in nursing or midwifery), and four participants (0.54%) did not declare their education.

### 3.2. Comparability of the Studied Groups

To verify the assumption that internal medicine nurses, surgical nurses and midwives do not differ in terms of sensitivity to justice, moral feelings and perceived stress at work, a series of one-factor ANOVA analysis of variance was conducted with the quality factor of the department (surgical, internal medicine, midwifery) and the dependent variable in terms of subscale scores: the Feelings in Moral Situations Scale, the Justice Sensitivity Inventory, and the Perceived Stress at Work Scale. The groups did not differ (p at least greater than 0.126) on any score of the mentioned scales and individual subscales. Justice sensitivity, from the perspective of the victim, witness and unauthorized beneficiary, was moderate. In contrast, from the perspective of the perpetrator, respondents reacted with high sensitivity (the lower limit of the score range). The frequency of experiencing the listed situations was moderate. The Feelings in Moral Situations Scale showed that respondents’ feelings were mostly at a moderate level. Perceived stress at work was also moderate. Thus, the groups can be considered comparable in terms of sensitivity to justice, moral feelings and in terms of perceived stress at work. For this reason, any possible differences between groups on the Questionnaire of Attitudes of Healthcare Workers towards Reporting of Clinical Adverse Events (P-RoCAES) should not be attributable to differences between groups in this regard (Appendix A).

### 3.3. Attitudes towards Adverse Events among Internal Medicine Nurses, Surgical Nurses and Midwives

Table 1 shows a comparison of the means in terms of the total score and each subscale of the P-RoCAES questionnaire between the three professional groups. For this purpose, a series of one-factor ANOVA analyses of variance was conducted, where the grouping variable was the type of department, and the dependent variable was the total score on the P-RoCAES questionnaire and on its individual subscales. While the overall score of the P-RoCAES (as well as on the each subscales) did not differ between internal medicine nurses, surgical nurses, and midwives, three significant group effects were observed on the subscales: guilt, perception of colleagues’ expectations, and transparency of procedures. Post hoc tests showed that in terms of: (1) feelings of guilt—internal medicine nurses scored higher compared to midwives (*p* = 0.011) and surgical nurses (*p* = 0.059); (2) perceived expectations of colleagues—internal medicine nurses scored higher compared to surgical nurses (*p* = 0.037); (3) transparency of procedures—surgical nurses scored higher compared to midwives (*p* = 0.08).

### 3.4. Relationship between Attitudes towards Adverse Events and Seniority, Age and Education of Respondents

First, the relationship with length of service and scores on the P-RoCAES questionnaire and individual subscales was examined. Significant correlations (r-Spearman) were obtained for the following: (a) feelings of guilt (r(686) = 0.15; *p* = 0.001); (b) transparency of procedures (r(686) = 0.12; *p* = 0.001); (c) benefits of adverse event reporting (r(686) = −0.08; *p* = 0.037). As seniority increased, scores suggesting positive attitudes in the subscales, i.e., guilt and transparency of procedures, increased, while scores suggesting negative attitudes toward perceived benefits of adverse event reporting decreased (although significant, the correlation is small).

Second, a series of one-factor ANOVA analyses of variance were conducted, where the grouping variable was the age group (21–30, 31–40, 41–50, 51–60) and the dependent variable was the total score on the P-RoCAES questionnaire and its individual subscales. Those in the 61–65 age group were excluded from the analyses due to the small size of the group (N = 24). Post hoc tests showed that, in general, scores suggesting positive attitudes in the mentioned subscales increased with age (Table 2).

Finally, the analyses discussed in the previous section of the text were repeated for the education grouping variable (bachelor’s, master’s, diploma). Post hoc tests showed that on the P-RoCAES questionnaire (total score) and its subscales—feelings of guilt, perceived criteria for identifying adverse events, perceived expectations of colleagues—respondents with a higher degree were the most positive, had the best understanding of the importance of criteria for identifying events, and had the highest perceived expectations of colleagues. Those with a nursing/midwifery diploma significantly had the lowest attitudes towards perceived benefits of adverse event reporting (Table 2).

## 4. Discussion

In the present study, internal medicine nurses, surgical nurses, midwives showed the following. (1) They did not differ in terms of justice sensitivity, moral feelings and perceived stress at work, although post hoc tests showed that in terms of the following: feelings of guilt—internal medicine nurses scored higher than midwives and surgical nurses; perceived expectations of colleagues—internal medicine nurses scored higher than surgical nurses; transparency of procedures—surgical nurses scored higher than midwives. (2) They showed a fairly high overall level of positive attitudes towards reporting clinical adverse events, (3) The older they were in age, the more positive they were about the importance in practice of transparent procedures, about the perceived benefits of adverse event reporting, and the higher feelings of guilt they had. (4) The longer they worked, the higher were the feelings of guilt they had, the more positive attitudes about the importance in practice of transparency of procedures they had, but they had negative attitudes about the benefits of adverse event reporting. (5) Those with a master’s degree (regardless of the type of department) had significantly higher positive overall attitudes, significantly higher feelings of guilt, as well as the best understanding of the importance of the criteria for identifying adverse events, the greatest perception of the expectations of colleagues and the benefits of adverse event reporting. There are a few research results on the attitudes of medical professionals, including nurses and midwives towards adverse events, using standardized survey tools, but a complete lack in linking them to morality and exposure to stress. As for attitudes, on the other hand, the results are inconclusive. In Polish studies, attitudes towards reporting adverse events tended to be negative, although internal medicine nurses showed slightly less reluctance to report, compared to nursing students [16,20]. In contrast, English studies have shown that physicians have less positive attitudes towards reporting adverse events, compared to nurses [17]. German studies, on the other hand, have not reported an exact response rate, due to the heterogeneity of professions and work experiences [18]. A study among Chinese nurses, similar to Polish nurses, showed that the attitude of those reporting adverse events was related to demographic characteristics such as education, length of service and job title [25,26,27]. Interestingly, the RoCAES questionnaire was used to evaluate the effectiveness of the educational measures taken on adverse event reporting. Improvements in all dimensions of the RoCAES scale were found as a result of training [28]. A report of a nationwide survey of the opinions of medical and nursing staff (3410 participants) on adverse event reporting and the requirements that reporting systems in healthcare should have, conducted in 2015 using a postal survey technique, shows that ward managers demonstrated high acceptance of an adverse event reporting system. They overwhelmingly (85%) agreed that its introduction would improve patient safety. Three out of four (78.3%) further stated that their motivation regarding reporting would be greater if the lessons learned were used not only on their ward [29]. According to Leńczuk-Gruba et al. [13], in order for positive changes to occur in terms of reducing the occurrence of adverse events, nurses and midwives should report all incidents they have perpetrated or witnessed. On the other hand, it is up to management to create an environment and atmosphere in medical institutions where those who report negligence and medical errors feel understood and personally safe. In the study presented here, midwives and nurses working in surgical and internal medicine units with longer length of service declared negative attitudes towards the perceived benefits of adverse event reporting, which may indicate that they feared reporting such incidents in the workplace or considered the reporting and monitoring system to be ineffective in daily practice, requiring corrective action.

According to the World Health Organization (WHO), the main obstacle to moving to a safer health care system is changing the culture from blaming people for mistakes to one in which mistakes are seen as an opportunity to improve the system and prevent adverse events. A strong safety culture encourages all members of the healthcare team to identify and reduce risks to patient safety by reporting errors and near misses, enabling root cause analysis and removing identified risks from the system. In a poorly defined and implemented safety culture, staff often hide mistakes out of fear or shame. Nurses and midwives have traditionally been trained to believe that clinical excellence is achievable and that “good” nurses/midwives do not make mistakes. Mistakes are seen as caused by carelessness, inattention, indifference or uninformed decisions. According to this approach, changes are needed, starting with assessing the phenomenon and then altering the narrative and encouraging the promotion of even more positive attitudes toward reporting clinical adverse events. Promoting a non-punitive response to adverse event reporting can improve nurses’ safety attitudes [30,31].

In conclusion, it should be noted that the presented work has its advantages and disadvantages/limitations. The former include the fact that an attempt was made to present the attitudes of nurses working in internal medicine, surgical wards, and as midwives towards the reporting of clinical adverse events using a standardized survey instrument, with a relatively large sample. The survey is characterized by such limitations as sampling by grade, an unequal sample size, and a limited study area, but covering about 40% of working nurses and midwives from 9 of the 12 existing hospitals in a major provincial city. The declarative responses of respondents and the impact of the social approval variable should be taken into account, as it is imbued in tools measuring feelings and moral behavior, where observational studies would be more valuable. Follow-up studies on a more diverse or representative sample may be needed to confirm findings, if generalizability is the goal.

### Implications for Practice

Shaping a culture of safety in health care (including the reporting of adverse events) can be influenced by individual attitudes. Feeling of guilt can motivate corrective action and be a constructive source of attitude formation. Investment should be made in educational programs aimed at the younger generation of nurses and midwives, without a master’s degree. Adverse event reporting should be systemic.

## 5. Conclusions

Based on the research material presented and the analysis of the results, the following can be concluded:

- the attitudes of the internal medicine nurses, surgical nurses and midwives towards reporting of clinical adverse events in P-RoCAES were positive, especially those with a master’s degree,
- respondents with a master’s degree had the best understanding of the importance of the criteria for identifying incidents and they also had the highest perception of the expectations of colleagues and the benefits of reporting adverse events (P-RoCAES subscales),
- in terms of guilt (P-RoCAES subscale), the longer employment tenure and having a master’s degree were associated with a higher sense of guilt and also the internal medicine nurses had higher guilt feelings compared to midwives and surgical nurses,
- the longer respondents worked, the more positive the attitude became towards the importance in practice of transparency in procedures, but there was a negative attitude towards the benefits of adverse event reporting (P-RoCAES subscales).

Individual attitudes can influence the development of a culture of safety in health care (including adverse event reporting). Guilt can be a positive motivational resource to change nurses’ and midwives’ attitudes towards adverse events. This is because guilt is triggered by failure caused by our actions. It arises when we transgress social norms, when there is a threat to our relationships with others, and when we harm someone or cause some loss. Guilt can have the function of protecting relationships between people and evoking caring. It can motivate corrective action, to pay attention to other people. It can be a constructive source of attitude change because it acknowledges our imperfections but does not condemn us for them. If it is felt excessively, without corrective action being taken, and it can lead to a significant lowering of mood and lower self-esteem [22]. Investment should be made in educational programs aimed at the younger generation of nurses and midwives, without a master’s degree, to raise awareness of the benefits of achieving better clinical adverse event reporting through higher education, continuing professional development. Adverse event reporting should be systemic, as the lack of perception of the benefits of reporting by surveyed nurses and midwives with longer seniority may signal a deficiency in the system and should result in corrective action.

## Figures and Tables

**Table 1:** Means and standard deviations of the P-RoCAES questionnaire and its subscales among surgical nurses, internal medicine nurses and midwives.

Questionnaire P-RoCAES	Surgical Nurses	Internal Medicine Nurses	Midwives	ANOVA Test
Total score	71.10 (7.60)	72.04 (7.99)	71.26 (7.25)	*F*(2.741) = 1.14, *p* = 0.319
Feeling of guilt	16.60 (3.13) ^A^	17.12 (3.11) ^A^^,B^	16.32 (2.74) ^B^	*F*(2.741) = 4.13, *p* = 0.017
Perceived criteria for identifying adverse events	17.14 (2.70)	17.35 (2.80)	17.84 (2.38)	*F*(2.741) = 2.45, *p* = 0.087
Perceived expectations of colleagues	17.56 (2.38) ^D^	18.02 (2.44) ^D^	17.60 (2.37)	*F*(2.741) = 2.93, *p* = 0.054
Perceived benefits of adverse event reporting	14.08 (2.18)	14.02 (2.35)	14.16 (2.04)	*F*(2.741) = 0.19, *p* = 0.830
Transparency of procedures	5.72 (1.05) ^C^	5.54 (1.24)	5.34 (1.16) ^C^	*F*(2.741) = 3.56, *p* = 0.029

Comments: Standard deviations (SD) are given in parentheses. Means with the same letter (e.g., ^B vs. B, C vs. C, D vs. D etc.^) within a row, are significantly different from each other (*p* < 0.05). ^A^ *p* = 0.059. The higher the scores, the more positive are attitudes towards reporting clinical adverse events. Color has been used to highlight the significant values.

**Table 2:** Means and standard deviations of the P-RoCAES questionnaire and its subscales for different age and education groups of respondents.

Questionnaire P-RoCAES	Age Group					Education			
	21–30	31–40	41–50	51–60	ANOVA Test	Bachelor’s Degree	Master’s Degree	Diploma	ANOVA Test
Total score	71.21 (7.31)	71.75 (8.83)	71.59 (7.60)	71.83 (7.42)	*F*(3.716) = 0.24, *p* = 0.867	71.05 ^A^ (6.95)	72.69 ^A,B^ (8.18)	70.13 ^B^ (7.86)	*F*(2.737) = 6.38, *p* = 0.002
Feeling of guilt	16.34 ^A,B^ (3.08)	16.40 ^C,D^ (3.54)	17.18 ^A,C^ (2.76)	17.22 ^B,D^ (2.80)	*F*(3.716) = 4.63, *p* = 0.003	16.49 ^A^ (3.08)	17.10 ^A^ (3.12)	17.03 (2.77)	*F*(2.737) = 3.10, *p* = 0.045
Perceived criteria for identifying adverse events	17.28 (2.85)	17.56 (2.86)	17.38 (2.61)	17.31 (2.58)	*F*(3.716) = 0.31, *p* = 0.816	17.19 ^A^ (2.66)	17.64 ^A,B^ (2.82)	17.02 ^B^ (2.44)	*F*(2.737) = 3.32, *p* = 0.037
Perceived expectations of colleagues	17.93 (2.47)	17.96 (2.51)	17.67 (2.25)	17.77 (2.44)	*F*(3.716) = 0.58, *p* = 0.631	17.67 ^A^ (2.24)	18.13 ^A,B^ (2.44)	17.32 ^B^ (2.62)	*F*(2.737) = 6.05, *p* = 0.002
Perceived benefits of adverse event reporting	14.30 ^A,B^ (2.03)	14.28 (2.27)	13.87 ^B^ (2.25)	13.79 ^A^ (2.44)	*F*(3.716) = 2.56, *p* = 0.054	14.10 ^A^ (2.14)	14.27 ^B^ (2.26)	13.30 ^A,B^ (2.39)	*F*(2.737) = 8.45, *p* = 0.001
Transparency of procedures	5.35 ^A^ (1.21)	5.55 (1.18)	5.49 ^B^ (1.07)	5.73 ^A,B^ (1.24)	*F*(3.716) = 3.55, *p* = 0.014	5.60 (1.09)	5.56 (1.27)	5.46 (1.18)	*F*(2.737) = 0.50, *p* = 0.602

Comments: Standard deviations (SD) are given in parentheses. Means with the same letter (e.g., ^A vs. A, B vs. B, C vs. C, D vs. D etc.^) within a row, are significantly different from each other (*p* < 0.05). ^B^ *p* = 0.060. Color has been used to highlight the significant values.

## Data Availability

Data are available from the project manager.

## Appendix A

**Table A1:** Means and standard deviations in individual scales and subscales.

	Surgical Nurses	Internal Medicine Nurses	Midwives	ANOVA Test
**The Justice Sensitivity Inventory**				
The victim’s perspective	29.26 (10.52)	30.31 (9.97)	29.37 (8.92)	*F*(2.740) = 0.92, *p* = 0.401
Frequency of experiencing situations of being a victim	2.71 (1.24)	2.57 (1.27)	2.46 (1.01)	*F*(2.740) = 1.52, *p* = 0.220
A witness’s perspective	30.23 (9.88)	31.35 (9.70)	30.11 (7.86)	*F*(2.740) = 1.34, *p* = 0.261
Frequency of experiencing situations of being a witness	2.71 (1.17)	2.72 (1.21)	2.57 (1.10)	*F*(2.740) = 0.84, *p* = 0.433
The unauthorized beneficiary’s perspective	30.70 (8.80)	30.70 (10.03)	30.43 (9.01)	*F*(2.740) = 0.04, *p* = 0.962
Frequency of experiencing situations of being an unauthorized beneficiary	2.13 (1.13)	2.14 (1.14)	2.32 (1.05)	*F*(2.740) = 1.27, *p* = 0.280
The perpetrator’s perspective	36.32 (9.58)	35.28 (11.44)	35.26 (10.92)	*F*(2.740) = 0.59, *p* = 0.552
Frequency of experiencing situations of being a perpetrator	2.00 (1.24)	2.04 (1.29)	2.03 (1.21)	*F*(2.740) = 0.07, *p* = 0.929
**The Feelings in Moral Situations Scale**				
Exceeding norms positive feelings	7.40 (5.25)	7.34 (5.46)	7.66 (5.21)	*F*(2.740) = 0.16, *p* = 0.850
Exceeding norms distancing	8.68 (3.92)	8.31 (4.29)	8.53 (4.05)	*F*(2.740) = 0.54, *p* = 0.582
Exceeding norms fear of punishment	12.71 (4.88)	12.75 (5.33)	12.63 (5.23)	*F*(2.740) = 0.03, *p* = 0.971
Exceeding norms shame	12.56 (5.10)	12.41 (4.93)	12.62 (4.69)	*F*(2.740) = 0.11, *p* = 0.895
Exceeding norms global guilt	17.96 (7.05)	18.78 (7.49)	18.78 (7.76)	*F*(2.740) = 0.78, *p* = 0.458
Exceeding norms feeling of repentance	15.57 (4.95)	16.14 (4.90)	15.78 (5.07)	*F*(2.740) = 0.91, *p* = 0.404
Realizing norms negative feelings	8.86 (5.93)	8.98 (6.30)	9.87 (6.40)	*F*(2.740) = 1.12, *p* = 0.328
Realizing norms hope/prize	13.70 (5.94)	13.55 (5.54)	14.56 (5.36)	*F*(2.740) = 1.53, *p* = 0.217
Realizing norms conceit	8.67 (6.20)	8.47 (5.79)	9.71 (6.02)	*F*(2.740) = 2.08, *p* = 0.126
Realizing norms pride	18.14 (6.14)	18.61 (5.73)	18.63 (5.75)	*F*(2.740) = 0.43, *p* = 0.650
Realizing norms certainty of rules	18.05 (6.36)	18.08 (5.53)	17.53 (6.01)	*F*(2.740) = 0.44, *p* = 0.646
**The Perceived Stress at Work Scale**				
Stress scale	21.80 (5.53)	21.97 (4.83)	21.97 (4.02)	*F*(2.740) = 0.08, *p* = 0.920

Comment: The table shows means, standard deviations (SD), F-values ANOVA test, *p*—significance level.
